# NK Cell IL-10 Production Requires IL-15 and IL-10 Driven STAT3 Activation

**DOI:** 10.3389/fimmu.2019.02087

**Published:** 2019-09-04

**Authors:** Sarah E. Clark, Kristina S. Burrack, Stephen C. Jameson, Sara E. Hamilton, Laurel L. Lenz

**Affiliations:** ^1^Department of Immunology and Microbiology, University of Colorado School of Medicine, Aurora, CO, United States; ^2^Department of Laboratory Medicine and Pathology, Center for Immunology, University of Minnesota, Minneapolis, MN, United States

**Keywords:** NK cell, IL-10, *Stat3*, IL-15, IL-15 complex, *Listeria (L.) monocytogenes*, *Plasmodium*, cerebral malaria

## Abstract

Natural killer (NK) cells can produce IFNγ or IL-10 to regulate inflammation and immune responses but the factors driving NK cell IL-10 secretion are poorly-defined. Here, we identified NK cell-intrinsic STAT3 activation as vital for IL-10 production during both systemic *Listeria monocytogenes* (Lm) infection and following IL-15 cytokine/receptor complex (IL15C) treatment for experimental cerebral malaria (ECM). In both contexts, conditional *Stat3* deficiency in NK cells abrogated production of IL-10. Initial NK cell STAT3 phosphorylation was driven by IL-15. During Lm infection, this required capture or presentation of IL-15 by NK cell IL-15Rα. Persistent STAT3 activation was required to drive measurable IL-10 secretion and required NK cell expression of IL-10Rα. Survival-promoting effects of IL-15C treatment in ECM were dependent on NK cell *Stat3* while NK cell-intrinsic deficiency for *Stat3, Il15ra*, or *Il10ra* abrogated NK cell IL-10 production and increased resistance against Lm. NK cell *Stat3* deficiency did not impact production of IFNγ, indicating the STAT3 activation initiated by IL-15 and amplified by IL-10 selectively drives the production of anti-inflammatory IL-10 by responding NK cells.

## Introduction

Natural killer (NK) cells are innate lymphoid cells (ILCs) that contribute to immunity through direct lysis of tumor or virus infected cells and though the secretion of immune-regulatory cytokines. The overall impact of NK cells in a given setting depends on their activation state and which cytokines they produce. In a number of animal infection models, NK cell secretion of IFNγ peaks within the first day of infection and promotes inflammatory responses that can contribute to pathogen clearance (1–7). IL-10 is also produced by both human and mouse NK cells in response to diverse microbial stimuli (8–13). IL-10 secretion potently limits host protection during Lm, *Leishmania*, and *Toxoplasma* infections (14–16), presumably through inhibitory effects on recruitment or activation of inflammatory myeloid cells (14). By contrast, the immune-dampening effects of NK cell IL-10 production are protective in the context of experimental cerebral malaria (ECM) (8). Cerebral malaria is a lethal complication from *Plasmodium* infection. There are no treatments available for cerebral malaria beyond anti-malarial drugs, for which resistance continues to rise (17). Strategies to manipulate NK cell IL-10 production could thus prove useful in treatment of diverse infectious diseases.

IL-12 is known to induce NK cell IL-10 secretion through activation of signal transducer and activator of transcription (STAT)-4 (16, 18). This pathway was further suggested to contribute to IL-10 production by NK cells responding to *Toxoplasma gondii* infection (16). However, IL-12/STAT4 signaling is not required for NK cell IL-10 secretion in the context of *Listeria monocytogenes* (Lm) bacteria or murine cytomegalovirus (MCMV) infections (19, 20). In the context of Lm infection, IL-18 production by a *Batf3*-dependent cell population was shown to promote NK cell IL-10 production (19, 20). However, IL-12 and IL-18 both also contribute to NK cell IFNγ production (19, 21, 22). To date, specific drivers of NK cell IL-10 production have not been identified.

Recent work showed that NK cells can also be driven to produce IL-10 in mice treated with an immune modulatory cytokine complex consisting of IL-15 bound to an IL-15Rα-Fc fusion protein (8). This IL-15C complex and similar cytokine complexes have been shown to improve anti-tumor immunity in mice, and several are in clinical trials for cancer immunotherapy (23–25). In the present study, we observed that IL-15 or IL-15C treatment could induce the activation of STAT3 in NK cells. STAT3 is known to orchestrate diverse cellular responses that include cell differentiation, proliferation, and inflammation. STAT3 has also previously been implicated in a coordinated “anti-inflammatory response” that is associated with IL-10 production (26). Prior work investigated the impact of *Stat3* deficiency restricted to NK cells and showed that this increased expression of granule enzymes and enhanced NK cell cytotoxicity (27). Correspondingly, mice with NK cell-restricted *Stat3* deficiency showed improved clearance of B16F10 melanoma (27). However, the impact of STAT3 activation on NK cell cytokine production has not been previously investigated.

Here, we generated conditionally-mutant mice lacking expression of Stat3 and other factors selectively in NK cells and used these to demonstrate an essential requirement for STAT3 activation in the induction of NK cell IL-10 production during both Lm infection and IL-15C treatment. Our data indicate that IL-15 signaling induces early STAT3 activation to initiate IL-10 production. In the context of Lm infection, this involves the capture or presentation of IL-15 by NK cell-expressed IL-15Rα. Subsequently, IL-10 feeds back through IL-10R to promote sustained STAT3 activation that drives NK cell IL-10 production. NK cell *Stat3* deficiency did not impact production of IFNγ, suggesting that STAT3 activation induced and sustained by IL-15 and IL-10 selectively drives NK cell production of IL-10. This pathway of NK cell IL-10 production is shown to be critical for regulating immune responses and host resistance during both Lm and *Plasmodium* infections.

## Materials and Methods

### Animals

Existing mouse strains used in this study included C57BL/6J (WT/B6), B6. *il10*^−/−^, B6. *Il10*-GFP reporter (Vert-X and Tiger) (28, 29), IL-15Rα floxed (30), Ncr1^cre^ (31), STAT3 floxed (32), and IL-10Rα floxed (33). In addition, Ncr1^cre^ × STAT3^fl/fl^ (NKSTAT3^−^), Ncr1^cre^ × IL-15Rα^fl/fl^ (NK15Ra^−^), and Ncr1^cre^ × IL-10Rα^fl/fl^ (NKIL10R^−^) were generated in our colony at the University of Colorado. All mice were purchased or generated on a C57BL/6J background. Adult male and female mice were used at 8–12 weeks of age. Mice were maintained in specific pathogen-free conditions in the University of Colorado Office of Laboratory Animal Resources and the University of Minnesota Research Animal Resources vivariums.

### Infections

For Lm infections, strain 10403S (originally obtained from Daniel Portnoy) was thawed from frozen stocks for dilution in tryptic soy broth (MP Biomedicals) supplemented with streptomycin (50 μg/mL) for growth to log phase. Bacteria grown to log phase were diluted in PBS and a sublethal dose of 10^4^ CFU was injected into the lateral tail vein i.v. Organs harvested for CFU enumeration were homogenized in 0.02% Nonidet P-40 with a tissue homogenizer (IKA Works). Serial dilutions were plated on trypticase soy broth (TSB) agar plates supplemented with streptomycin (50 μg/mL) and grown overnight at 37°C.

For PbA infections, *Plasmodium berghei* ANKA was passaged *in vivo*, and parasitized RBCs (pRBCs) were used to create stocks frozen in Alsever's solution and glycerol (9:1 ratio) for storage in liquid nitrogen until use as previously described (8). Freshly thawed stocks of pRBCs were used for infection of experimental mice. Mice were infected with 1 × 10^6^ PbA pRBCs in PBS into the lateral tail vein i.v. and survival was monitored.

### Cytokine Complex Treatments

The cytokine complex IL-15C was prepared by combining 0.75 μg rIL-15 (eBioscience) with 7 μg rIL-15Rα-FC chimera protein (R&D) and incubating for 20–30 min at 37°C. IL-2C was prepared by combining 1.0 μg carrier-free recombinant mouse rIL-2 (eBioscience) with 10 μg αIL-2 Ab S4B6 (Bio X Cell). Mice were treated with cytokine complexes by injection into the lateral tail vein i.v. In IL-10 GFP reporter mice, STAT3 inhibitor (NSC 74859, Tocris) was resuspended in DMSO and diluted in PBS. Mice were treated with 5 μg/g as previously described (34).

### Cell Isolation and Stimulations

NK cells were purified by negative selection from the spleens of mice using the EasySep Mouse NK cell Isolation Kit (StemCell Technologies). Purified NK cell populations were >80% NK1.1+CD3– cells. For NK cell culture stimulations, rIL-2 and rIL-12 were added at a concentration of 50 pg/mL and supernatants were collected at 72 h. For NK cell culture stimulations for immunoblots, rIL-15 or rIL-10 100 pg/mL were added for 15 min prior to cell collection for lysate preparation.

For co-culture experiments, B6. *il10*^−/−^ BMDCs were cultured in GM-CSF for 6 d prior to plating overnight in 24 well plates at a concentration of 3 × 10^5^ BMDCs (>90% CD11c+) per well. For L1S stimulations, 10 ng/mL LPS (L8274 Sigma-Aldrich) together with 30 μg/mL purified L1S protein were added to BMDCs for 1 h. For Lm infections, log phase Lm was added at a multiplicity of one bacterium per BMDC. Cells were washed at 1 h and gentamycin was added to media at 10 μg/mL. Purified NK cells were added to cultures 2 h after L1S + LPS treatment or Lm infection at a ratio of 1:10 (NK cells:BMDCs). Where indicated, a STAT3 inhibitor (stattic, Sigma-Aldrich) was added to co-cultures at a concentration of 40 ug/mL. Supernatants were harvested for analysis of IL-10 at 72 h using an ELISA kit (BD Biosciences).

For detection of IL-10 in IL-15C treated mice, whole splenocytes were harvested into HBSS media and single cell suspensions were prepared by passing tissue through a 70 μM strainer followed by removal of red blood cells with an RBC lysis buffer (0.15 M NH_4_Cl, 10 mM KHC0_3_, 0.1 mM Na_2_EDTA, pH 7.4). Cells from the blood were collected in HBSS plus cations and heparin (Sigma-Aldrich) prior to RBC lysis. 10^6^ cells (>95% CD45+) from the spleen and blood were plated per well in a 96 well plate and incubated at 37°C + CO_2_ for 18 h. Supernatants were collected for detection of IL-10 by ELISA.

### Flow Cytometry

Spleens were harvested into HBSS plus cations media (Invitrogen) containing 1 mg/mL type 4 collagenase (Worthington) and incubated for 30 min at 37°C. Single cell suspensions were obtained following passage through a 70 μM strainer. Red blood cells were removed by treatment with RBC lysis buffer. Cell suspensions were incubated with anti-CD16/32 (2.4G2 hybridoma supernatant) for 30 min to block Fc receptors. Staining was completed in FACS buffer (1% BSA, 0.01% NaN3, PBS) and fixed in 1% paraformaldehyde. Antibodies for staining included anti-CD3 (clone 1452C11), NK1.1 (clone PK136), NKp46 (clone 29A1.4), CD45.2 (clone 104), Ly6C (clone KH1.4), CD11b (clone M1/70), CD27 (clone LG.7F9), anti-TCRβ (clone H57-597), anti-p-STAT5 (clone 47/pY694), anti-p-STAT3 (clone 4/pY705), CD49b (clone HMalpha2), and IL-10Rα (CD210, clone 1B1.3a). All antibodies were purchased from eBioScience, BioLegend, or BD Biosciences. For p-STAT analysis, cells were stained for surface markers, fixed in 1.5% formaldehyde, permeabilized using methanol, and stained for p-STAT3 and p-STAT5. For IL-10 (GFP) analysis in Vert-X reporter mice, RBCs were lysed using ACK lysis buffer then stained for surface markers. For Tiger reporter mice, cells were treated with saponin for intracellular staining and IL-10-gfp signal was amplified by staining with a rabbit monoclonal anti-GFP followed by goat anti-rabbit IgG Alexa Fluor 488 (Life Technologies). A minimum of 100,000 events per sample were collected on an LSRII (BD Biosciences) using BD FACSDiva and FlowJo software (Tree Star Technologies) was used for data analysis.

### Western Blotting

Equivalent cell lysates were prepared for western blotting from purified NK cells and loaded onto a 10% SDS-PAGE gel under reducing conditions. Protein was transferred to nitrocellulose membranes following semi-dry transfer. Membranes were blocked in Odyssey Blocking Buffer (LI-COR) and probed with primary antibodies against p-STAT3 (Y705, clone D3A7), total STAT3 (clone 79D7), and β-actin (clone 8H10D10) (Cell Signaling) followed by secondary antibodies against rabbit or mouse IgG. Protein expression was detected on an Odyssey imaging system (LI-COR) and images were analyzed with Image Studio software.

### Quantitative Real-Time PCR

A quantitative PCR machine (Bio-Rad) was used to detect expression of *Stat3, il10, il15Ra, gapdh*, and *hmbs* transcripts from cDNA samples prepared from RNA using reverse transcription and RNA extraction kits (Bio-Rad). Primers for transcript detection included STAT3F: CTGTAGAGCCATACACCAAGCAGCAGC and STAT3R: GGTCTTCAGGTACGGGGCAGCAC (27), IL-10F: AGGGTTACTTGGGTTGCCAA and IL-10R: CACAGGGGAGAAATCGATGA (35), IL-15RaF: GCCTCAAGTGCATCAGAGACC and IL-15RaR: ACCTTTGGTGTCACTACTGTTGGC (36), GAPDHF: ATGTTCCAGTATGACTCCACTCAC and GAPDHR: GAAGACACCAGTAGACTCCACGACA, HMBSF: GAGTCTAGATGGCTCAGATAGCATGC and HMBSR: CCTACAGACCAGTTAGCGCACATC (37).

### Study Approval

These studies were approved by the Animal Care and Use Committee (protocol #00313) and the Institutional Biosafety Committee of the University of Colorado School of Medicine as well as the Institutional Animal Care and Use Committee (protocol #1705-34830A) of the University of Minnesota.

### Statistical Analysis

Graphing and statistical analysis were conducted using Prism (GraphPad) software. Statistical tests included *t*-tests, ANOVA and log-rank (Mantel-Cox). *p* < 0.05 was considered significant.

## Results

### STAT3 Activation Is Associated With NK Cell IL-10-Production

**Figure 1:**
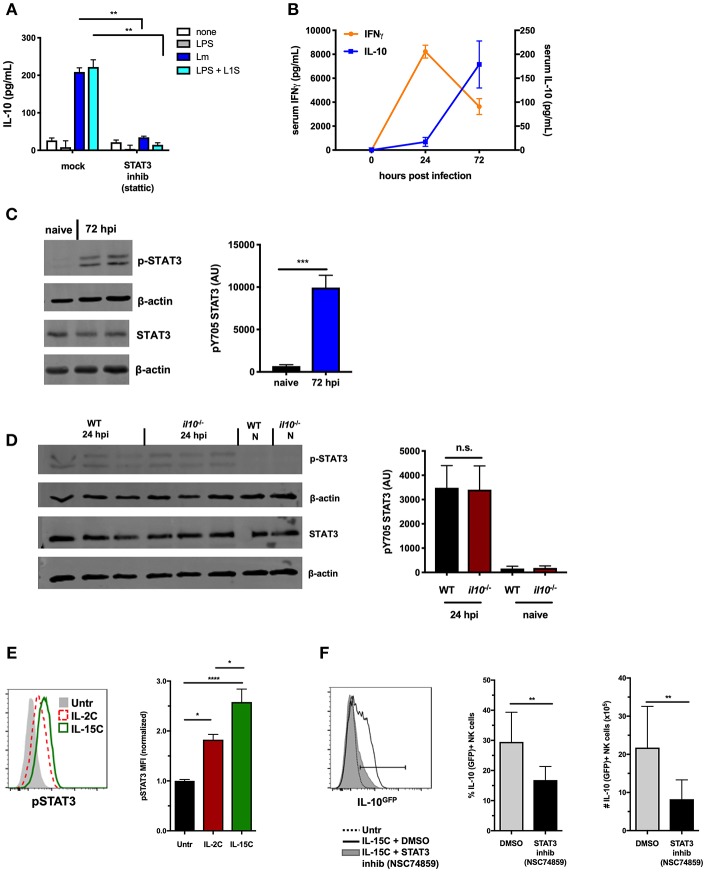
STAT3 activation is associated with NK cell IL-10-production. **(A)** Supernatant IL-10 at 72 h after co-culture with B6. *il10*^−/−^ BMDC. BMDC were infected with Lm or stimulated with LPS + L1S for 1 h before addition of purified WT NK cells ± STAT3 inhibitor (Stattic). **(B)** Serum IFNγ and IL-10 at indicated time points from WT mice infected with 10^4^ Lm i.v. **(C)** Immunoblot detection of p-STAT3 (Y705) and total STAT3 from lysates of purified NK cells isolated from WT naive mice and 72 hpi with 10^4^ Lm i.v. Densitometry quantification shown as Arbitrary Units (AU) normalized to β-actin loading controls. **(D)** Detection of p-STAT3 (Y705) and total STAT3 from NK cells isolated from naïve (N) or 24 hpi with 10^4^ Lm i.v. WT and B6. *il10*^−/−^ mice. **(E)** Expression of p-STAT3 measured by flow cytometry in splenic NK cells (NK1.1+NKp46+TCRβ-) from B6. *il10*^−/−^ untreated mice compared to mice treated (on d0 and d2) with IL-2C or IL-15C i.v. Splenocytes were harvested 1 h following d2 treatment. Histogram and mean fluorescent intensity (MFI) are shown. MFI values normalized relative to naive mice. **(F)** Percentage and number of IL-10-GFP+ NK cells (NK1.1+NKp46+TCRβ-) from IL-10-GFP reporter mice treated with IL-15C i.v. on d0 and d2 and three treatments of either STAT3 inhibitor (NSC 74859) or DMSO i.p. on d0, d2, and d3. Splenocytes were harvested on d5 for analysis of IL-10-GFP expression. ^*^p < 0.05, ^**^p < 0.01, ^***^p < 0.001, ^****^p < 0.0001 as measured by *t* test.

Our prior studies showed Lm infection or products induced NK cells to secrete IL-10 (14). This response is independent of IL-12 or STAT4 and instead requires IL-18 and at least one other DC product (19). Toward identifying this factor, we evaluated signaling pathway(s) required for NK cell IL-10 production in response to Lm or Lm-derived stimuli. Using a previously-described cell co-culture model system (14, 21), negatively sorted splenic NK cells from wildtype B6 mice were cultured 72 h with bone marrow-derived dendritic cells (BMDCs) from B6. *Il10*^−/−^ mice. When BMDCs were infected with Lm or stimulated with a recombinant protein corresponding to the “L1S” region of the Lm p60 virulence protein (plus a priming agent such as LPS) they induced IL-10 production from the purified NK cells (Figure 1A). This NK cell IL-10 production was found to be significantly reduced in co-cultures treated with a small molecule inhibitor of STAT3 activation and dimerization, stattic (38).

Systemic Lm infection elicits an early increase in serum IFNγ and a delayed production of IL-10 (Figure 1B). NK cells contribute to both responses (14). We thus utilized the Lm infection model to further address the potential involvement of STAT3 activation in regulation of NK cell IL-10 secretion. Splenic NK cells were isolated by negative selection at both 24 and 72 h post-infection (hpi) and evaluated for activation of STAT3. Tyrosine (Y705) phosphorylated STAT3 (p-STAT3) was detectable in lysates of these NK cells at both time points after infection (Figures 1C,D), though more p-STAT3 was observed at 72 hpi. IL-10 can bind IL-10R to elicit p-STAT3 (39), and thus likely contributes to the increased p-STAT3 seen at 72 hpi. However, NK cells produce little or no IL-10 at 24 hpi and little serum IL-10 is detected at this early time point (14). This suggested another factor might be responsible for inducing p-STAT3 at 24 hpi (Figure 1D). Indeed, the amount of p-STAT3 at 24 hpi was comparable in NK cells isolated from B6 and B6. *Il10*^−/−^ mice. Thus, STAT3 activation occurs in NK cells throughout Lm infection, and early p-STAT3 activation is independent of IL-10.

NK cell IL-10 production can also be induced by treatment of mice with a cytokine/cytokine receptor complex comprised of IL-15 and IL15Rα (IL-15C) (8). The kinetics of NK cell IL-10 production are similar in mice treated with IL-15C and during systemic Lm infection. IL-15 is known to bind IL-2/IL-15Rβ and common gamma chain (γc) R subunits to activate STAT5 and STAT3 in appropriate contexts (40, 41). We thus asked if NK cell IL-10 production in mice treated with IL-15C might also be associated with STAT3 activation. To test this and eliminate the potential impact of IL-10, phosphorylation of intracellular STAT5 and STAT3 was evaluated in NK cells from spleens of B6. *Il10*^−/−^ mice following i.v. treatment with IL-15C or IL-2C (a control cytokine/cytokine receptor complex that does not drive NK cell IL-10 production (8). Staining intensity for p-STAT3 expression was significantly higher in NK cells from IL-15C treated mice compared with IL-2C-treated and untreated mice (Figure 1E), while p-STAT5 staining was similar in NK cells from mice treated with IL-2C or IL-15C (Supplemental Figure 1A). These results demonstrate that IL-15C treatment activates STAT3 in NK cells independent of IL-10 production.

The possible requirement for STAT3 activation in the induction of IL-10 production was tested using B6. *Il10*-GFP reporter (Vert-X) mice (29) treated with IL-15C in the presence or absence of the STAT3 inhibitor NSC74859 (34). Inhibitor treatment significantly reduced the percentage and number of IL-10 (GFP+) NK cells responding to IL-15C treatment at 72 h (Figure 1F). Treatment of mice with IL-15C has also been reported to drive expansion of NK cells (25, 42). Consistent with the interpretation that STAT3 drives this expansion, numbers of NK cells were reduced in the spleens of mice treated with both IL-15C and the STAT3 inhibitor vs. IL-15C alone (Supplemental Figure 1B). The STAT3 inhibitor did not significantly alter NK cell maturation as defined by surface expression of CD27 and CD11b (Supplemental Figure 1C). These data together demonstrate an early and sustained STAT3 activation that correlates with NK cell IL-10 production in two distinct models: Lm infection and IL-15C treatment.

### NK Cell-Intrinsic STAT3 Is Required for the IL-10 Response Induced by IL-15C Treatment

**Figure 2:**
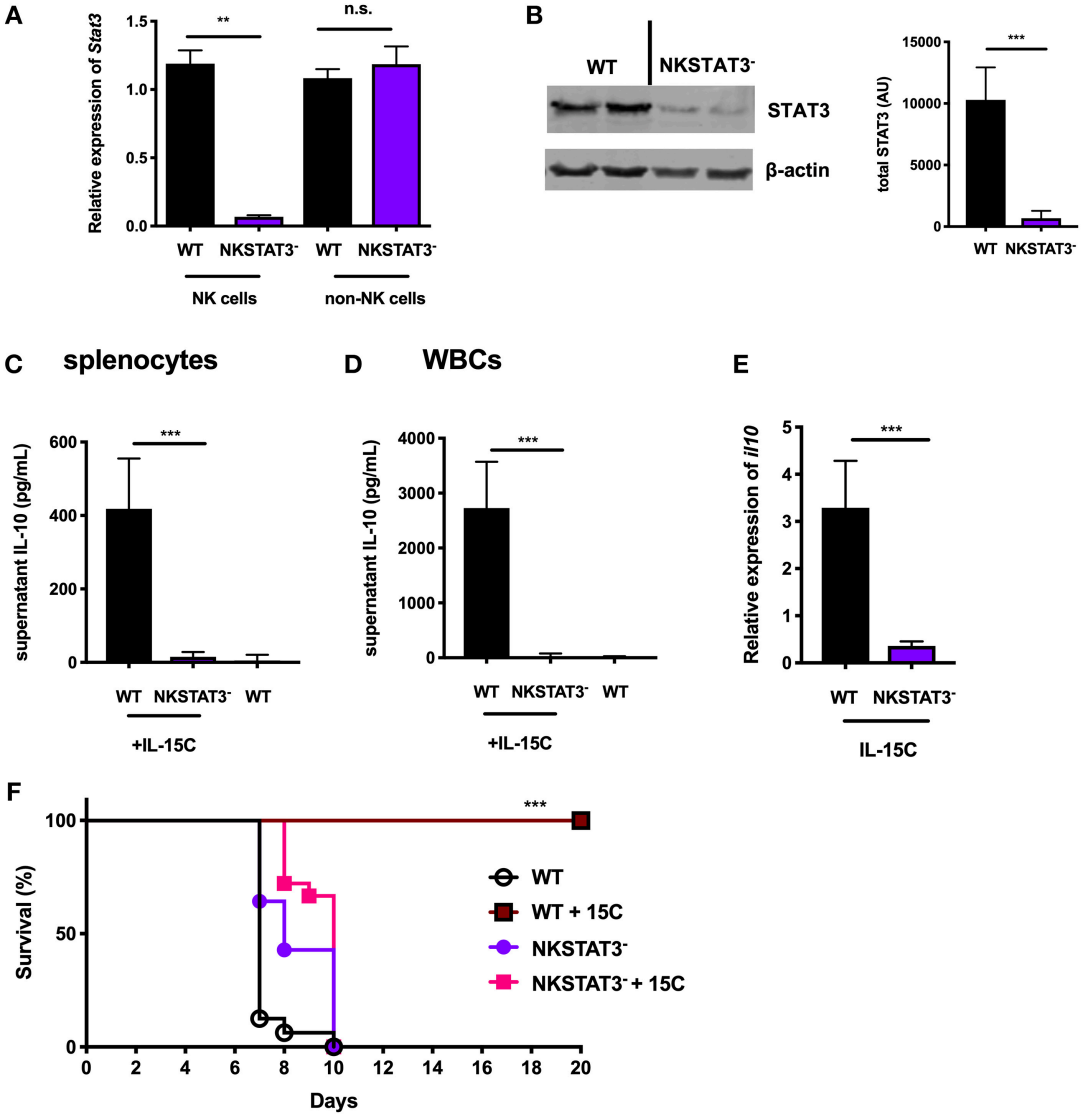
NK cell-intrinsic STAT3 is required for the IL-10 response induced by IL-15C treatment. **(A)** qRT-PCR detection of *Stat3* expression (relative to *hmbs* and *gapdh*) and **(B)** immunoblot detection of total STAT3 in purified NK cells or remaining non-NK cell population from WT and NKSTAT3^−^ naïve mice. Densitometry shown as Arbitrary Units (AU) normalized to β-actin loading controls. **(C)** Supernatant IL-10 in RBC-lysed whole splenocyte and **(D)** white blood cell (WBC) (>95% CD45+) 18 h cultures from WT untreated mice and WT or NKSTAT3^−^ mice treated with IL-15C at days−5 and−3. **(E)** qRT-PCR detection of *il10* expression (relative to *hmbs* and *gapdh*) in equivalent numbers of purified NK cells isolated from WT or WT or NKSTAT3^−^ mice treated with IL-15C at days−5 and−3. **(F)** Survival of WT or NKSTAT3^−^ mice treated with IL-15C or PBS on days 2 and 3 post infection with 1 × 10^6^ PbA parasitized red blood cells i.v. ^**^p < 0.01 as measured by *t* test. ^***^p < 0.001 as determined by log-rank (Mantel-Cox) test.

To evaluate whether intrinsic STAT3 is required for NK cell IL-10 secretion, we generated mice with conditional STAT3 deficiency in NK cells by crossing *Stat3*^fl^ (floxed *Stat3*) (32) and *Ncr1*^*cre*^ (31) mice. The splenic NK cell population remained intact in the *Ncr1*^*cre*^ × *Stat3*^*fl*/*fl*^ (NKSTAT3^−^) mice, with equivalent percentages and numbers of NK cells and similar distribution of maturation markers as seen in WT control mice (Supplemental Figures 1D,E). In contrast to non-NK cells, little *Stat3* transcript was detected in purified splenic NK cells from NKSTAT3^−^ mice (Figure 2A). Similarly, NK cells purified from NKSTAT3^−^ mice had significantly reduced levels of STAT3 protein compared to those from WT mice (Figure 2B). We thus treated groups of control and NKSTAT3^−^ mice with IL-15C and evaluated IL-10 production. Total splenocytes and white blood cells (>95% CD45^+^) were harvested and cultured to evaluate secretion of IL-10 protein (Figures 2C,D). IL-10 protein was readily detected in culture supernatants from IL-15C-treated WT mice, but little or no IL-10 secretion was detected in cultures from untreated mice or NKSTAT3^−^ mice treated with IL-15C. To confirm NK cells were responsible for the observed *Stat3*-dependent IL-10 production, RNA was also isolated from equivalent numbers of purified splenic NK cells from IL-15C-treated WT and NKSTAT3^−^ mice and *il10* transcripts were quantified by qRT-PCR. The expression of *il10* was significantly reduced in NK cells from NKSTAT3^−^ mice (Figure 2E). IL-15C treatment also selectively failed to increase splenic NK cell numbers in NKSTAT3^−^ mice (Supplemental Figure 1F). These data indicate that both IL-10 production and NK cell expansion in response to IL-15C treatment are dependent on NK cell-intrinsic STAT3 signaling.

Treatment with IL-15C protects against fatal malaria disease in a mouse model of cerebral malaria known as ECM, and this therapeutic effect is dependent on IL-10 production by NK cells (8). We therefore evaluated the impact of NK cell-intrinsic STAT3 deficiency on IL-15C therapeutic efficacy during ECM. Mice were infected with *Plasmodium berghei* ANKA (PbA) parasitized red blood cells and treated with IL-15C at days 2 and 3 post-infection. IL-15C treatment protected WT mice against ECM as judged by increased survival relative to control (PBS) treated mice (Figure 2F). In contrast, NKSTAT3^−^ mice succumbed to infection regardless of receiving IL-15C treatment (Figure 2F). These findings together demonstrate that NK cell-intrinsic STAT3 is required for NK cell IL-10 production and the protective effects associated with IL-15C therapeutic treatment in mice with cerebral malaria.

### Intrinsic STAT3 Is Required for the NK Cell IL-10 Response During Systemic Lm Infection

**Figure 3:**
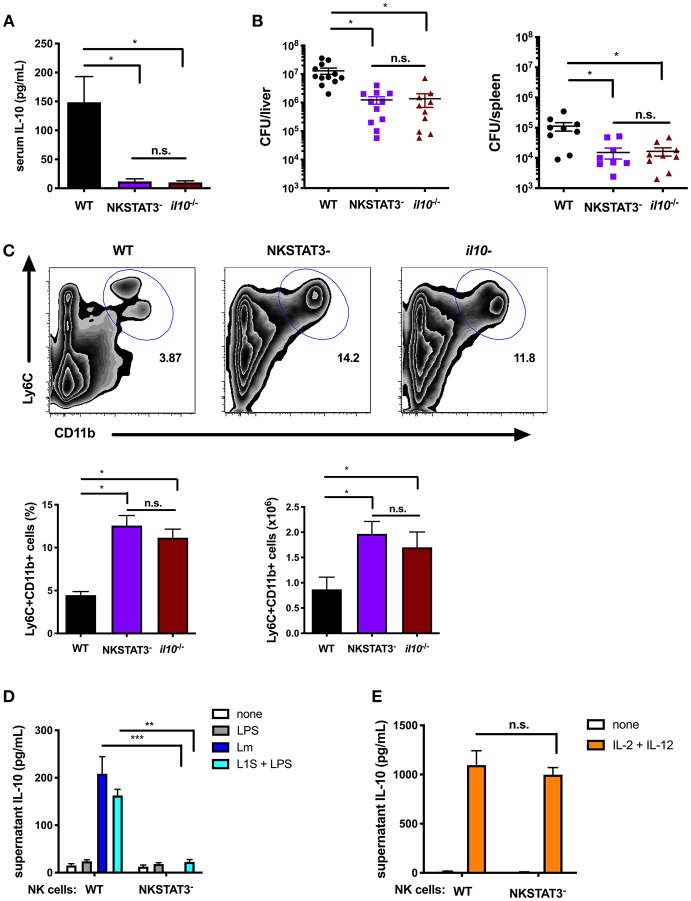
Intrinsic STAT3 is required for the NK cell IL-10 response during systemic Lm infection. **(A)** Serum IL-10 at 72 hpi with 10^4^ Lm i.v. from WT, NKSTAT3^−^, and B6. *il10*^−/−^ mice. **(B)** Lm burdens shown as colony forming units (CFU) per liver and spleen detected from WT, NKSTAT3^−^, and B6. *il10*^−/−^ mice at 72 hpi with 10^4^ Lm i.v. **(C)** Representative dot plot, percentage, and cell number of Ly6C+CD11b+ cells from total splenocytes isolated from WT, NKSTAT3^−^, and B6. *il10*^−/−^ mice at 72 hpi with 10^4^ Lm i.v. **(D)** Supernatant IL-10 at 72 h following B6. *il10*^−/−^ BMDC 1 h infection with Lm or activation with LPS + L1S and co-cultured with NK cells purified from WT or NKSTAT3^−^ mice. **(E)** Supernatant IL-10 at 72 h following stimulation of NK cells purified from WT or NKSTAT3^−^ mice with rIL-2 + rIL-12 (50 pg/mL). ^*^p < 0.05, ^**^p < 0.01, ^***^p < 0.001 as measured by *t* test.

To investigate the impact of NK cell STAT3 during bacterial infection, NKSTAT3^−^ mice were infected i.v. with 10^4^ CFU Lm. The increased serum IL-10 observed at 72 hpi in B6 control mice was not seen in NKSTAT3^−^ mice, which produced no more IL-10 than infected B6. *Il10*^−/−^ mice (Figure 3A). Consistent with the detrimental effects of NK cell IL-10 production in this model, Lm burdens in the livers and spleens of NKSTAT3^−^ mice were significantly reduced at 72 hpi and equivalent to those seen in B6. *Il10*^−/−^ mice (Figure 3B). Thus, the selective loss of STAT3 in NK cells had a similar impact on bacterial burdens as the complete absence of IL-10. Recruitment of inflammatory myeloid cells to the spleens of Lm-infected mice is also increased in the absence of IL-10 and was similarly increased in Lm-infected NKSTAT3^−^ and B6. *Il10*^−/−^ mice (Figure 3C). The recruitment of these cells in NKSTAT3^−^ mice correlates with improved resistance to Lm, as it does in mice lacking either IL-10 or NK cells (14, 19). In contrast to IL-15C treatment, Lm infection does not induce NK cell proliferation. Thus, we did not observe reduced splenic NK cell numbers in the Lm infected NKSTAT3^−^ spleens (Supplemental Figure 1G). Rather, NK cell numbers were modestly increased in the mice selectively lacking NK cell *Stat3* or lacking all IL-10 (Supplemental Figure 1G). Type 1 ILC also express *Ncr1*/NKp46 (43, 44), thus deletion of *Stat3* in this population might also have impact on the response to Lm infection. To determine the potential contribution of ILC1 to IL-10 production during Lm infection, we used CD49b to distinguish between traditional NK cells (CD49b+) and ILC1 (CD49b–) (44). Gating on GFP+ cells from 3 dpi B6. *Il10*-GFP reporter (Tiger) mice revealed that CD49b+ traditional NK cells were overwhelmingly the most abundant IL-10-producing NK1.1+CD3– population (Supplemental Figure 1H). Likewise, gating on CD49b– ILC1 cells revealed that only a minor subset of the gated population became GFP+ in response to Lm (Supplemental Figure 1I). These data demonstrate that there is a minimal contribution of ILC1 to overall IL-10 production during Lm infection, arguing that the deletion of *Stat3* in ILC1 cells has minimal impact on production of IL-10 and thus is unlikely to contribute to the significant reductions in IL-10 and increase pathogen burdens in the NKSTAT3- mice. Hence, *Stat3* expression is vital for NK cell production of IL-10. Further, this IL-10 production suppresses myeloid cell recruitment to sites of infection and correlates with increased host resistance to systemic Lm.

IL-12 and STAT4 are implicated in the regulation of NK cell IL-10 production during certain parasitic infections (16, 18), but are not required for this response during Lm infection (19). Consistent with these prior results, we confirmed that NK cell *Stat3* was required for IL-10 release in co-cultures with Lm-infected or L1S + LPS stimulated BMDCs (Figure 3D), but found purified NK cells from NKSTAT3^−^ mice produced IL-10 equivalently as those from WT mice when cultured with IL-2 and IL-12 (Figure 3E). These findings argue that STAT3 acts independently from IL-12/STAT4 to drive IL-10 production in NK cells exposed to distinct stimuli.

### IL-15 *cis*-Presentation Contributes to STAT3 Activation Required for NK Cell IL-10 Production During Lm Infection

**Figure 4:**
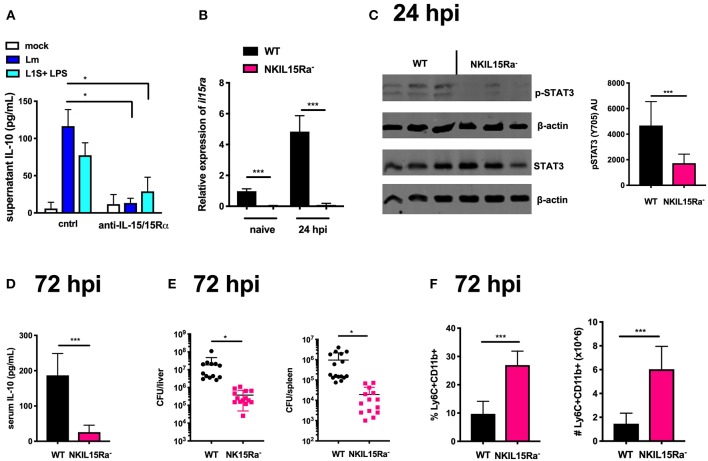
IL-15 *cis*-presentation contributes to STAT3 activation and NK cell IL-10 production during Lm infection. **(A)** Supernatant IL-10 at 72 h following il10^−/−^ BMDC 1 h infection with Lm or activation with LPS + L1S and co-cultured with NK cells purified from WT mice in the presence or absence of anti-IL-15/IL-15Rα. **(B)** qRT-PCR detection of *il15r*α expression (relative to *hmbs* and *gapdh*) from purified NK cells from WT or NKIL15R^−^ naïve mice or 24 hpi with 10^4^ Lm i.v. **(C)** Immunoblot detection of p-STAT3 (Y705) and total STAT3 from purified NK cells isolated from WT or NKIL15Ra^−^ mice isolated at 24 hpi with 10^4^ Lm i.v. Densitometry shown as Arbitrary Units (AU) normalized to β-actin loading controls. **(D)** Serum IL-10 and **(E)** Lm burdens shown as CFUs per organ from WT or NKIL15Ra^−^ mice at 72 hpi with 10^4^ Lm i.v. **(F)** Percentage and cell number of Ly6C+CD11b+ cells from total splenocytes isolated from WT or NKIL-15Ra^−^ mice at 72 hpi with 10^4^ Lm i.v. ^*^p < 0.05, ^***^p < 0.001 as measured by *t* test.

Considering the results above using IL-15C, we tested and observed that recombinant IL-15 induced STAT3 phosphorylation in NK cells purified from naïve B6 mice (Supplemental Figure 2A). Given the short *in vivo* half-life of soluble IL-15, it is usually found membrane-bound to producer cells or in a high affinity complex with IL-15Rα (45, 46). Thus, to test if IL-15 might impact IL-10 production in NK/BMDC co-cultures, we used an antibody that neutralizes the biological activity of IL-15/15Rα. This neutralization significantly reduced supernatant IL-10 in co-cultures infected with Lm or treated with L1S + LPS (Figure 4A). Testing the impact of IL-15 on NK cell activation *in vivo* is complicated by the fact that IL-15/IL-15Rα expression and *trans*-presentation by accessory cells is necessary for NK cell development (47, 48). However, recent experiments using mixed bone marrow chimeras demonstrated NK cells express IL15Rα and can present IL-15 in *cis-* to promote NK cell activation (36). We thus generated mice with conditional NK cell deletion of IL-15Rα by crossing *Ncr1*^*Cre*^ mice with an *Il15ra*^*fl*^ (floxed) strain (30) and used these NK15Ra^−^ mice to ask how *cis*-presentation of IL-15/IL-15Rα affected NK cell activity during systemic Lm infection. To confirm the deletion, NK cells were isolated from spleens of naïve and Lm-infected B6 and NK15Ra^−^ mice. Quantitative RT-PCR demonstrated *Il15ra* expression in the B6 NK cells and showed this was ablated in the NK15Ra^−^ NK cells (Figure 4B). Importantly, the absence of NK cell *Il15ra* expression did not alter the development, maturation or survival of the NK15Ra^−^ NK cells as measured by cell numbers and expression of CD11b or CD27 (Supplemental Figures 2B,C). We next evaluated STAT3 phosphorylation in NK cells isolated at 24 or 72 hpi from spleens of Lm-infected NK15Ra^−^ or WT B6 mice. Similar numbers of NK cells were observed in both groups of mice throughout the infection (Supplemental Figures 2D,E). The abundance of total STAT3 was also similar in both groups of NK cells, but amounts of p-STAT3 were significantly reduced in the *Il15ra*-deficient NK cells at 24 hpi (Figure 4C) with a reduction also evident at 72 hpi (Supplemental Figure 2F). These data indicate that NK cell IL-15Rα expression contributes importantly to the STAT3 activation observed during Lm infection.

To further determine the consequence of NK cell-intrinsic IL-15Rα deficiency on the Lm-induced systemic IL-10 response, we quantified serum IL-10 and tissue bacterial burdens at 72 hpi. NK cell-restricted *Il15ra* deficiency profoundly reduced both systemic IL-10 and Lm burdens in the liver and spleen (Figures 4D,E). Consistent with our earlier observation that NK cell dependent systemic IL-10 suppresses recruitment of inflammatory myeloid cells (14, 19), there was a significant increase in these cell populations in the spleens of infected NK15Ra^−^ mice (Figure 4F). Thus, NK cell expression of *Il15ra* contributes to STAT3 activation and IL-10 production by NK cells during Lm infection, presumably by enhancing NK cell access to limited quantities of IL-15 available in the infected mice. These data further suggest that *cis*-presentation of IL-15/IL-15Rα activates STAT3 to promote NK cell IL-10 secretion during Lm infection (e.g., at/by 72 hpi).

### NK Cell-Intrinsic IL-10R Boosts STAT3 Activation and IL-10 Secretion During Lm Infection

**Figure 5:**
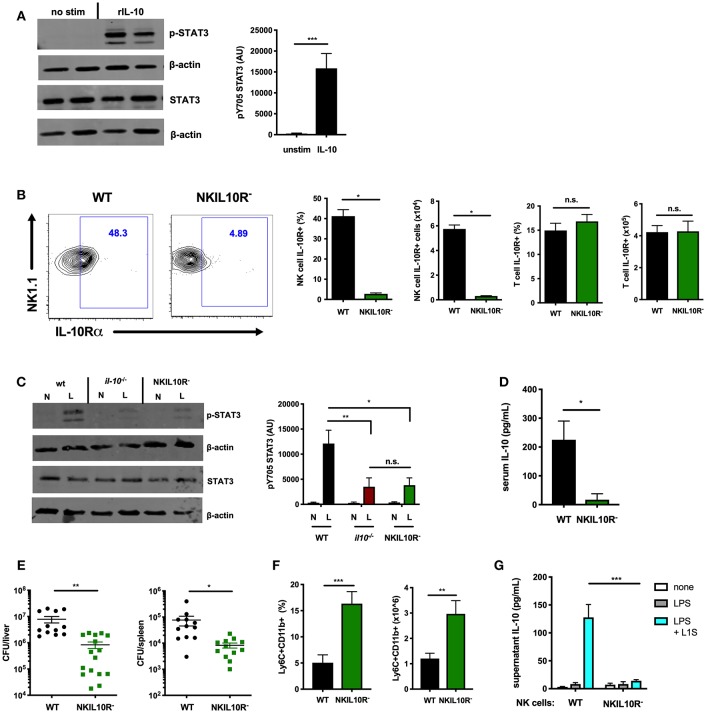
NK cell-intrinsic IL-10R boosts NK cell IL-10 secretion during Lm infection. **(A)** Immunoblot detection of p-STAT3 (Y705) and total STAT3 from lysates of purified NK cells isolated from the spleens of WT naïve mice following stimulation with rIL-10 (100 pg/mL) for 15 min. Densitometry shown as Arbitrary Units (AU) normalized to β-actin loading controls. **(B)** Representative flow cytometry plots and compiled percentage and cell numbers of IL-10Rα+ NK cells (NK1.1+CD3–) cells or T cells (NK1.1+CD3) from total splenocyte population isolated from either WT or NKIL10R- naïve mice. **(C)** Immunoblot detection of p-STAT3 (Y705) and total STAT3 from lysates of purified NK cells isolated from WT, B6. *il10*^−/−^, or NKIL10R^−^ naïve (N) mice or 72 hpi with 10^4^ Lm i.v. (L). Densitometry shown as Arbitrary Units (AU) normalized to β-actin loading controls. **(D)** Serum IL-10, **(E)** Lm burdens per organ, and **(F)** percentage and cell number of Ly6C+CD11b+ cells from total splenocytes isolated from WT or NKIL10R^−^ mice 72 hpi with 10^4^ Lm i.v. **(G)** Supernatant IL-10 at 72 h following B6. *il10*^−/−^ BMDC 1 h activation with LPS + L1S and co-cultured with NK cells purified from WT or NKSTAT3^−^ mice. ^*^p < 0.05, ^**^p < 0.01, ^***^p < 0.001 as measured by *t* test.

The data above implicate IL-15/IL15Rα in at least the initial activation of STAT3, but residual p-STAT3 was evident in NK cells from NKIL15Ra^−^ mice (Figure 4C and Supplemental Figure 2F). This led us to ask what other factors might be contributing to STAT3 activation and driving NK cell IL-10 production. IL-10 itself can signal to activate STAT3 in many cell types (39, 49). We found that recombinant IL-10 induced STAT3 phosphorylation in naïve splenic NK cells (Figure 5A), suggesting that IL-10R ligation on NK cells might act to enhance STAT3 activation and IL-10 secretion.

IL-10 activation of STAT3 requires ligation of a heterotetrameric IL-10R complex (26, 49). Thus, to test if IL-10-induced STAT3 activation might amplify IL-10 production from NK cells, we generated an additional mouse strain with NK cell-intrinsic IL-10R deficiency. The IL-10Rα subunit is uniquely used by IL-10 (50–52). Hence, *Ncr1*^cre^ and *Il10r1*^*fl*^ (floxed) (33) mice were crossed to generate the NKIL10R^−^ strain. IL-10Rα surface expression in these mice was lost on splenic NK cells but not T cells (Figure 5B). IL-10Rα deficiency did not noticeably affect NK cell development, as equivalent NK cell numbers and maturation subsets were present in the spleens of B6 and NKIL10R^−^ mice (Supplemental Figures 3A,B). We thus measured p-STAT3 in lysates of NK cells purified from naïve or Lm-infected WT, IL-10-deficient, and NKIL10R^−^ mice at 72 hpi. NK cell numbers remained comparable in the NKIL10R^−^ and WT mice at 72 hpi and the former cells remained negative for IL-10Rα staining (Supplemental Figures 3C,D). There was a dramatic increase in p-STAT3 in the infected WT mice, but the p-STAT3 response was similarly weak in NK cells from *Il10*^−/−^ and NKIL10R^−^ mice (Figure 5C). These data demonstrate the importance of IL-10 in boosting NK cell STAT3 activation during Lm infection but also confirm NK cells receive additional IL-10-independent signals that drive STAT3/IL-10 during *in vivo* Lm infection.

To evaluate the effects of NK cell IL-10R deficiency on susceptibility to infection, NKIL10R^−^ and WT mice infected with Lm as above were evaluated for IL-10 and bacterial burdens. Serum IL-10 was significantly reduced in the NKIL10R^−^ mice (Figure 5D). Lm burdens in the spleen and liver were also significantly lower in NKIL10R^−^ mice (Figure 5E), and more inflammatory myeloid cells accumulated in the spleens of these mice compared to the infected WT animals (Figure 5F). Finally, we observed that when co-cultured for 72 h with LPS and L1S-stimulated B6. *Il10*^−/−^ BMDCs, NK cells purified from NKIL10R^−^ spleens failed to produce IL-10 (Figure 5G). This contrasts with the measurable IL-10 in cultures using NK cells from WT mice. Hence, NK cell-intrinsic IL-10Rα is critical to boost STAT3 activation and IL-10 production in NK cells responding to Lm infection or Lm-derived stimuli.

### Requirement for NK Cell IL-10R Expression in the IL-10 Response to IL-15C Treatment

**Figure 6:**
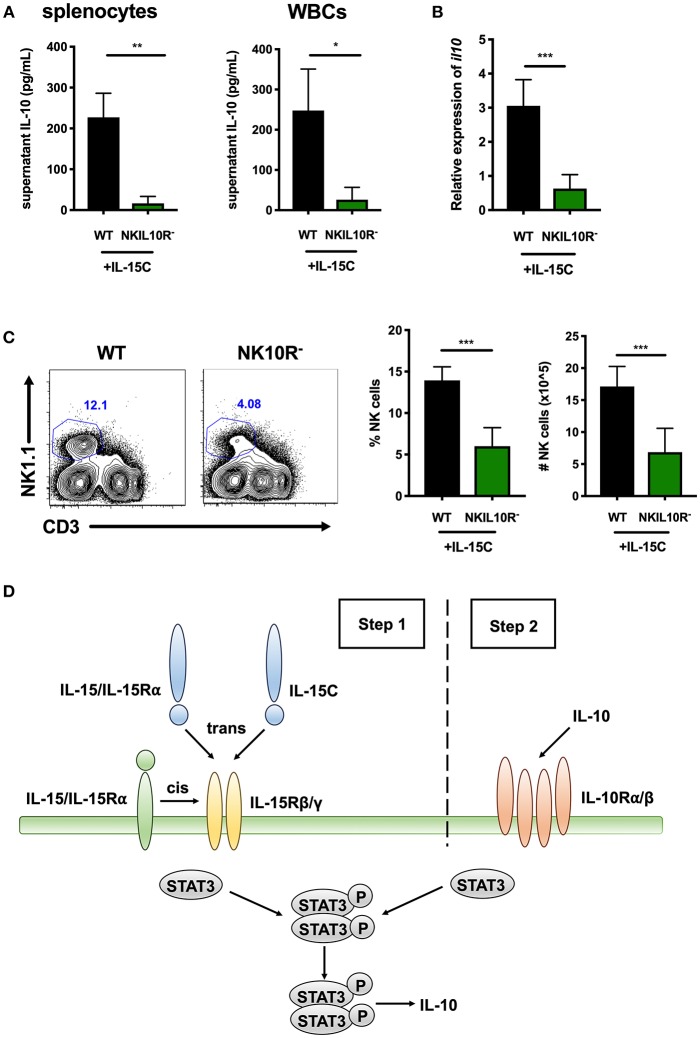
Requirement for NK cell IL-10R expression in the IL-10 response to IL-15C treatment. **(A)** Supernatant IL-10 in RBC-lysed whole splenocyte and white blood cell (WBC) (>95% CD45+) 18 h cultures from WT or NKIL10R^−^ mice treated with IL-15C i.v. at days−5 and−3. **(B)** qRT-PCR detection of *il10* expression (relative to *hmbs* and *gapdh*) in equivalent numbers of purified NK cells isolated from WT or NKIL10R^−^ mice treated with IL-15C i.v. at days−5 and−3. **(C)** Representative flow cytometry plots and compiled percentage and cell numbers of NK cells (NK1.1+CD3–) from WT or NKIL10R^−^ mice treated with IL-15C i.v. at days−5 and−3. **(D)** Model of STAT3-dependent NK cell production of IL-10 driven by initial IL-15 signaling (Step 1) followed by IL-10R feedback (Step 2) during both Lm infection and IL-15C treatment. ^*^p < 0.05, ^**^p < 0.01, ^***^p < 0.001 as measured by *t* test.

We further asked if NK cell IL-10R signaling might contribute to STAT3-dependent IL-10 production in mice treated with IL-15C. Here, WT and NKIL10R^−^ mice were treated with IL-15C as above. Splenic or blood leukocytes were harvested 72 h after the last IL-15C treatment and cultured overnight. Supernatant IL-10 concentrations were measured and observed to be significantly lower in cultures from NKIL10R^−^ compared to WT mice (Figure 6A). We next evaluated the abundance of *il10* mRNA in purified splenic NK cells from the IL-15C treated WT and NKIL10R^−^ mice. There was a significant reduction in *Il10* transcripts in the sorted NKIL10R^−^ NK cells compared to an equivalent number of WT B6 NK cells (Figure 6B). However, following IL-15C treatment we observed that splenic NK cell numbers were also lower in NKIL10R^−^ vs. WT mice (Figure 6C). This is consistent with the fact that IL-15C treatment expands NK cell numbers in spleens of WT, but not NKSTAT3^−^ mice (25, 42) (Supplemental Figure 1F). Since IL-15C is not known to signal directly through IL-10R, these findings suggest that feedback through NK cell IL-10R promotes both cell expansion and STAT3-dependent IL-10 secretion in mice responding to IL-15C.

These data together with results from the above studies support the model that during both Lm infection and IL15C treatment, IL-15 signals to NK cells to promote STAT3 activation that, perhaps in conjunction with other signals, acts to promote an initial wave of IL-10 secretion (Figure 6D, step 1). This NK cell-derived IL-10 then ligates IL10R on producer or bystander NK cells to amplify or sustain STAT3 activation and thus drive more robust IL-10 production (Figure 6D, step 2).

### STAT3 Activation in NK Cells Is Uniquely Required to Elicit Their Production of IL-10, but Not IFNγ

**Figure 7:**
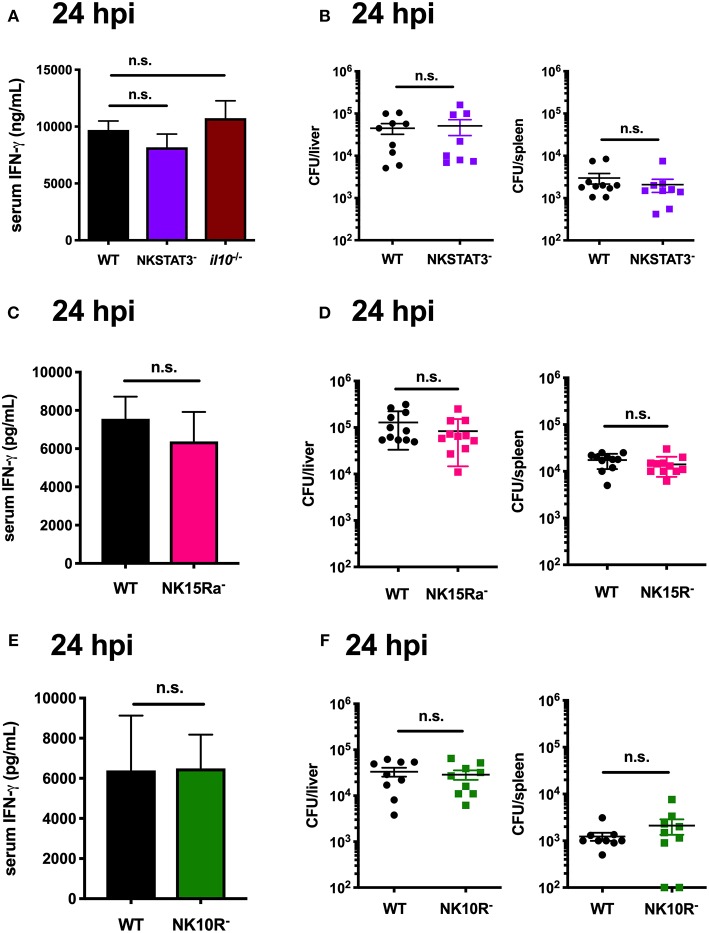
STAT3 activation in NK cells is uniquely required to elicit their production of IL-10, but not IFNγ. **(A)** Serum IFNγ and **(B)** Lm burdens per organ in WT, NKSTAT3^−^, or B6. *il10*^−/−^ mice at 24 hpi with 10^4^ Lm i.v. **(C)** Serum IFNγ and **(D)** Lm burdens per organ in WT or NK15Ra^−^ mice at 24 hpi. **(E)** Serum IFNγ and **(F)** Lm burdens per organ in WT or NK10R^−^ mice at 24 hpi.

The data above focused on IL-10 production by NK cells at 72 hpi. However, during Lm infection these cells are a major source of potentially anti-microbial IFNγ at 24 hpi (14, 53, 54). Production of IFNγ by NK cells is known to be driven by signals that include IL-12 and IL-18 (7, 54, 55). However, it remained possible that STAT3, IL-15Rα, and IL-10Rα signaling might also be important for the NK cell IFNγ response. To test this, we performed a series of experiments wherein matched groups of WT, NKSTAT3^−^, NKIL15Ra^−^, and NKIL10R^−^ mice were infected with Lm as above and sacrificed at the peak of NK cell IFNγ-production (24 hpi). Serum IFNγ and tissue Lm burdens were equivalent between the WT and mutant mice in each case, demonstrating that NK cell-intrinsic *Stat3, Il15ra*, and *Il10r1* do not affect IFNγ production but rather contribute selectively to the ability of NK cells to produce IL-10 during Lm infection (Figures 7A–F). These results provide additional evidence that NK cell IFNγ and IL-10 production are regulated by different stimuli, and suggest it may be possible to selectively interfere with signaling events to specifically manipulate NK cell pro- vs. anti-inflammatory cytokine responses.

## Discussion

STAT3 regulates a diverse repertoire of cellular responses and plays an important role in immunity to both cancer and infection. Here, we discovered an important and previously-unappreciated role of cell-intrinsic STAT3 in promoting NK cell IL-10 secretion in two distinct model systems: Lm infection and therapeutic treatment of mice with IL-15C. Our data demonstrated that STAT3 uniquely affects IL-10, but not IFNγ responses during Lm infection and further identified important drivers and boosters of the STAT3 activation during Lm infection and IL-15C treatment. The findings here have important implications for our understanding of IL-10 production by NK cells and thus their ability to dampen inflammatory responses during bacterial and parasite infections. Furthermore, our findings suggest that strategies targeting NK cell STAT3 could be used to selectively manipulate regulatory NK cell activity.

While our data implicate NK cell STAT3 as a key driver of IL-10 production, the precise mechanisms by which it promotes this response remain unclear. When phosphorylated, p-STAT3 can enter the nucleus to bind and induce transcription from the proximal *Il10* promoter in B and myeloid cell lines (56). In T cells STAT3, STAT4, and STAT5 also bind other regulatory regions in the *Il10* locus to recruit p300 histone acetylase to modify histones for increased *Il10* transcription (57). Since IL-15C treatment increases *il10* mRNA in NK cells (8), we speculate that activation by IL-15 also drives STAT3 recruitment to these loci in NK cells. Alternatively, activated STAT3 may act post-transcriptionally to increase *Il10* mRNA abundance, as was shown to occur in macrophages (58). This latter mechanism involves modification of tristetraprolin (TTP), a regulator of *il10* mRNA stability (59, 60). Whether TTP might also impact *il10* mRNA stability and IL-10 secretion in NK cells has not been investigated. We speculate that IL-15-driven STAT3 activation may act to promote initial *il10* transcription and the ensuing IL-10 production in turn promotes further IL-10 by enhancing the stability of *Il10* mRNA. By contrast, we observed that IL2C promotes STAT3 activation but fails to drive IL-10 secretion. In T lymphocytes, IL-2 stimulation elicits acetylation of STAT5, leading to blocked transcription of full-length *Stat5* (61). IL-2 may use a similar mechanism to prevent *Il10* transcription in NK cells.

Our Lm infection studies in mice with NK cell-intrinsic STAT3 deficiency showed that preventing STAT3 activation in this cell population has a protective effect. Similarly, inhibition of either STAT3 or IL-10Rα was recently shown to reduce lung burdens in a model of chronic infection with *Mycobacterium tuberculosis* (62). While this study did not address which cell types were essential for the observed therapeutic effects, the phenotype observed suggests STAT3-dependent IL-10 production similarly contributes to susceptibility in the context of this important pulmonary bacterial pathogen and demonstrate the potential for therapeutic targeting of STAT3/IL-10/IL-10R. We observed increased myeloid cell recruitment in the absence of NK cell-intrinsic STAT3. Likewise, reduced STAT3 activation in mice with a mutant *gp130* allele was shown to correlate with increased neutrophil recruitment in a model of peritoneal inflammation induced by *Staphylococcus epidermidis* (63). However, prior work suggested that loss of STAT3 in all hematopoietic cells increases susceptibility to Lm infection (64). Here, STAT3 supported early neutrophil mobilization that was thought to be driven by G-CSF and contribute to protection (65). Similarly, in mice with *Stat3* deficiency restricted to LysM+ neutrophils and macrophages susceptibility to peritonitis was increased in a cecal ligation and puncture (CLP) model (66). In this context, myeloid cell *Stat3* was further shown to be important for increased bactericidal activity of macrophages *in vitro* (66). In the context of the ECM model used here, we also observed a protective effect for NK cell STAT3, likely due to suppression of inflammation-driven tissue damage by IL-10 from NK cells. Overall, these data suggest that STAT3 function can play conflicting roles in different cell types and disease settings. This is consistent with observations of increased susceptibility to respiratory tract infections in humans with both gain-of-function and loss-of-function *Stat3* mutations (67, 68). Still, therapeutic approaches that inhibit or promote STAT3 activation in NK cells could prove useful to improve host resistance when used appropriately in bacterial and parasitic infections.

Studies here also defined stimuli that mediate STAT3 activation in NK cells. We observed that IL-15 can drive NK cell STAT3 activation in the context of both IL-15C treatment and Lm infection. The p-STAT3 in these contexts was seen with gated or purified NK cells very early (1 h) after IL-15C treatment and both early (24 h) and late (72 h) after systemic Lm infection. Association with IL-15Rα is important for stabilization of the otherwise labile IL-15 cytokine (45), which supports the use of IL-15/IL-15Rα-Fc (IL-15C) rather than IL-15 alone in therapeutic settings (23). The finding here that NK cell IL15Rα expression is important for IL-10 production during Lm infection suggests that expression of this receptor enables NK cells to compete more effectively for limited IL-15, but do not establish the source of IL-15 in this context. One possibility is that IL-15 or IL-15/IL-15Rα is directly extracted from the membrane of accessory cells such as DC, a model supported by data from a previous study (46). IL-15C treatment may similarly cause direct STAT3 activation in NK cells to promote their IL-10 production *in vivo*. However, it is important to emphasize that IL-15 is not sufficient to stimulate IL-10 production by cultured NK cells and there is instead a requirement for an additional factor(s) to drive this NK cell response (8). Indeed, p-STAT3 during Lm infection was still seen in NK cells from NKIL15Ra- mice, indicating other contributors to STAT3 activation in this setting.

Results from NKIL10R1^−^ mice indicated an important additional role for direct IL-10 stimulation in driving NK cell IL-10 secretion. IL-10 autocrine regulation through STAT3 has been previously demonstrated in other cell types, including macrophages, B cells and T cells (69–71). For B cell lymphoma, IL-6 and IL-10 feedback mediate STAT3 constitutive activation (72). In macrophages, Blimp-1 cooperates with IL-10 to amplify IL-10 production through STAT3 (70). The absence of suppressor of cytokine signaling 3 (SOCS3) binding to phosphorylated IL-10R, compared to the IL-6 receptor component gp130, may contribute to sustained IL-10R/STAT3 feedback (73). The sources of IL-10 that drive NK cell p-STAT3 during Lm infection and IL-15C treatment remain to be defined.

In the Lm infection model, IL-18 was also shown to be required for induction of NK cell IL-10 production (19). However, like IL-15, IL-18 failed to induce the NK cell IL-10 response by itself. IL-18 stimulation can also act in concert with IL-12 to boost NK cell IFNγ production (19, 74). Thus, IL-18 is appears to be an important driver of both pro- and anti-inflammatory NK cell activities. In contrast, our observations here indicate that NK cell-intrinsic deficiencies for *Stat3, Il15ra*, or *Il10r1* do not impact systemic IFNγ production or Lm bacterial burdens at 24 h post-infection. The lack of a requirement for these factors in IFNγ production is seen in spite of the fact that p-STAT3 activation is observed in NK cells at this early time point. These data are consistent with the model that a distinct set of regulatory factors drive NK cell production of pro-inflammatory IFNγ and anti-inflammatory IL-10. Hence, the IL-15R/STAT3/IL-10R pathway described here appears to be a unique driver of NK cell IL-10 production during bacterial infection. Previous results also suggest there is distinct regulation of NK cell IFNγ and IL-10 secretion in the IL-15C/PbA model (8). Thus, therapeutic modulation of STAT3 activation may be an effective strategy to selectively manipulate NK cell IL-10 production and its downstream impact on inflammatory and CD8+ T cells in diverse settings.

Therapeutic effects of IL-15C are observed in cancer and thought to depend on the expansion and activation of CD8^+^ T cells and NK cells (24, 25, 75). The importance of NK cells in this setting is supported by the finding that IL-15C improves tumor clearance in *Rag1*-deficient mice, which lack CD8^+^ T cells (76). However, NK cells in Rag1-deficient mice display hyperresponsiveness (77), and depletion of CD8+ T cells, but not NK cells, negates IL-15C-mediated tumor clearance (23). Results from prior studies further suggest STAT3 suppresses NK cell cytotoxicity (27). Hence, the observations here that IL-15C-drives STAT3 activation in NK cells and their production of IL-10 may indicate that targeting of NK cells in this context could reduce the efficacy of anti-cancer IL-15C therapy or at least limit NK cell cytotoxicity. It may therefore be worth testing if blockade of STAT3 activation in NK cells improves the efficacy of IL-15C in the context of cancer immunotherapy.

The use of *Ncr1*^cre^ to drive conditional deletion of *Stat3, Il15ra*, and *Il10r1* allowed dissection of the specific impact of these widely-expressed regulators in NK cells. These deletions clearly impacted the response of NK cells *in vitro* and *in vivo* in a manner that mimics effects previously seen in mice with NK cell depletion (8, 14). However, it is important to note that expression of *Ncr1*/NKp46, while primarily restricted to NK cells, has also been reported in non-NK ILC1 cells, intestinal epithelial ILC3 cells and *in vitro* expanded γδT cells (43, 44, 78, 79). Our findings indicate that ILC1 are not a major source of IL-10 and thus deletion of these factors in ILC1 likely has minimal impact on IL-10 production and its effects on host resistance to Lm or PbA. However, it remains to be seen if or how deletion of *Stat3, Il15ra*, or *Il10r1* in these other populations might impact resistance to other infections or otherwise alter inflammatory and immune responses. The development and application of more specific strategies for deleting or inactivating genes/gene products in NK or these other cell types would be required to fully address these issues in future work.

In summary, we have identified NK cell-intrinsic STAT3, IL-15Rα and IL-10R expression as critical and specific drivers of immune regulatory IL-10 production by this important innate lymphoid cell type. These factors and NK cell IL-10 secretion strongly impact immune responses dictating host survival in both systemic Lm infection and a model of cerebral malaria. Specifically, STAT3 signaling and NK cell IL-10 production is detrimental to host innate resistance to Lm, but promotes survival in a model of cerebral malaria. These results highlight disparate roles for STAT3 activation during the immune response to microbial infection, even within a single cell type. STAT3 inhibitors and IL-15C complexes are thus, respectively, attractive candidates for treatment of these infections. STAT3 inhibitors and IL-15C have also shown promise in therapeutic approaches to treat cancers. However, IL-15C-driven activation of STAT3 in NK cells could dampen NK cell cytotoxicity and or mediate other undesirable effects through the induction of IL-10. Recognizing these issues may lead to approaches to more selectively target NK and other immune cells for improved immune therapies of cancer and infections. Toward this goal it will also be important to further define the signaling pathways mediating NK cell IL-10 production during bacterial and other infections.

## Data Availability

All datasets generated for this study are included in the manuscript/Supplementary Files.

## Author Contributions

SC, KB, SJ, SH, and LL conceived and designed experiments. SC and KB performed the experiments. SC and LL wrote the paper. KB, SJ, and SH edited the manuscript.

### Conflict of Interest Statement

The authors declare that the research was conducted in the absence of any commercial or financial relationships that could be construed as a potential conflict of interest.
