# Supplementary material for: NK Cell IL-10 Production Requires IL-15 and IL-10 Driven STAT3 Activation
Source: Front Immunol. 2019 Sep 4;10:2087. doi: 10.3389/fimmu.2019.02087 (PMC6736993; doi:10.3389/fimmu.2019.02087)
Supplement: Supplementary file 1 [file Data_Sheet_1.pdf]

Supplemental Figure 1.

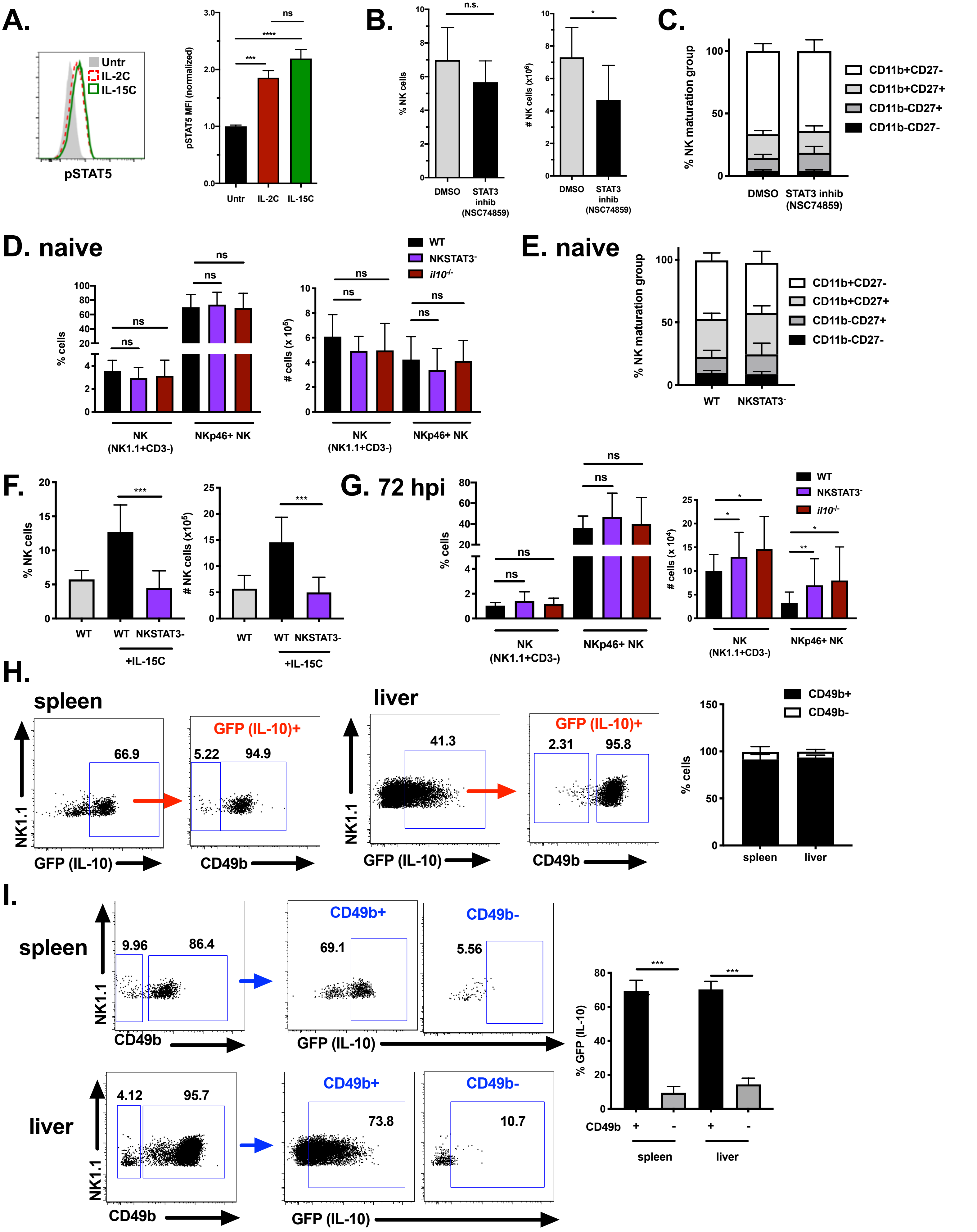

Supplemental Figure 2.

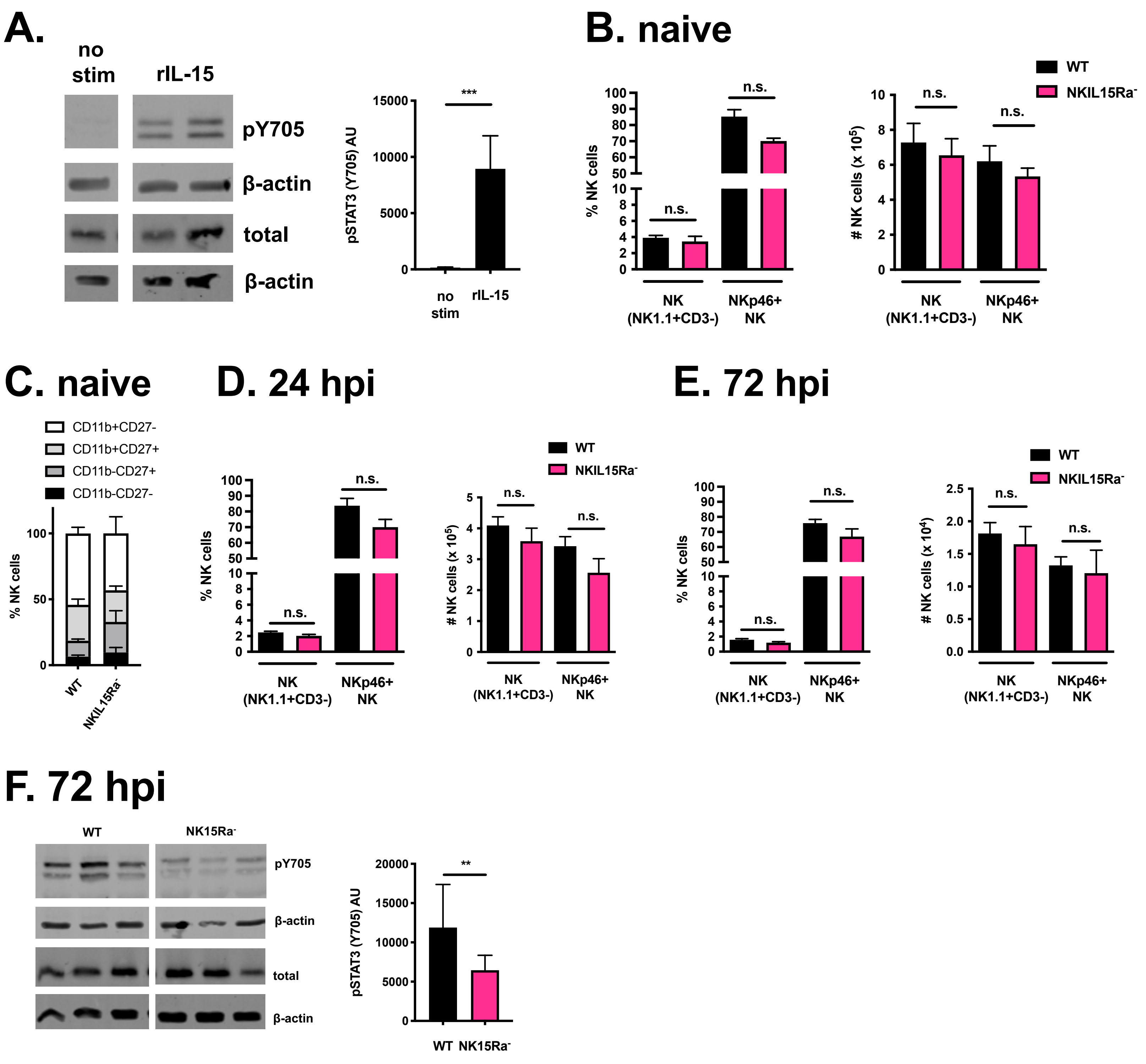

Supplemental Figure 3.

A. naive

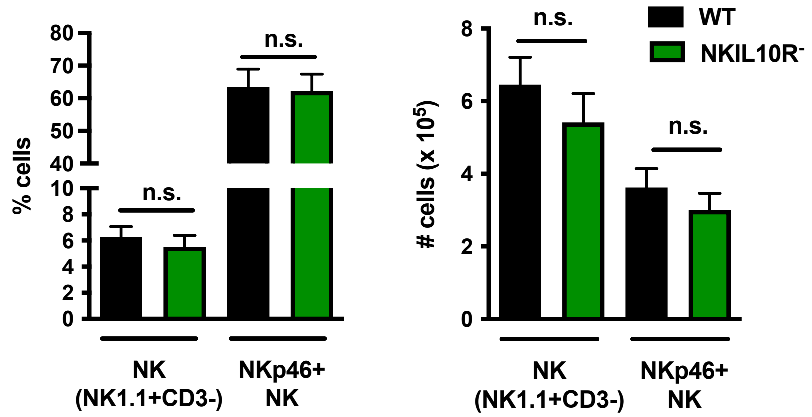

B. naive

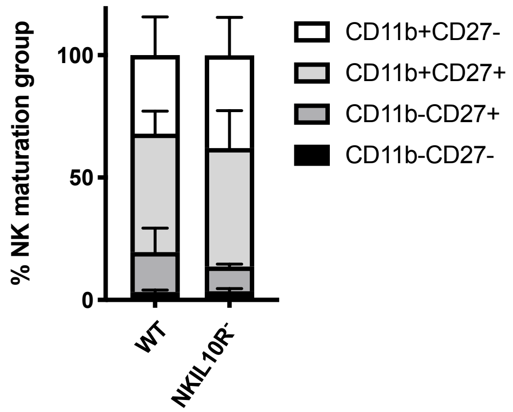

C. 72 hpi

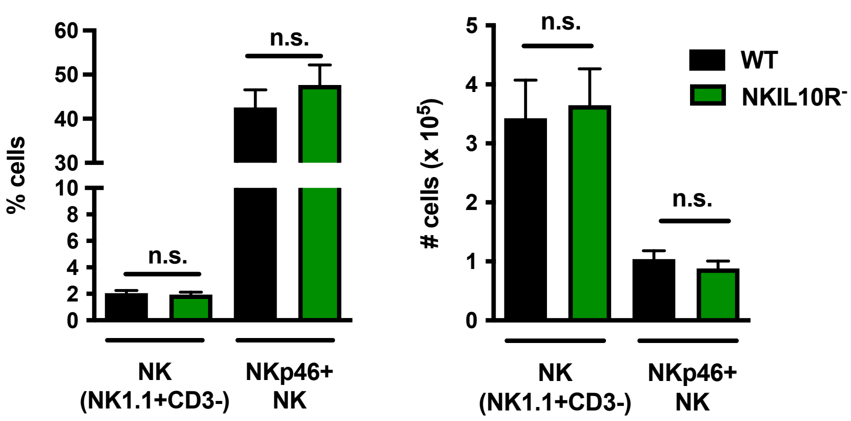

D. 72 hpi

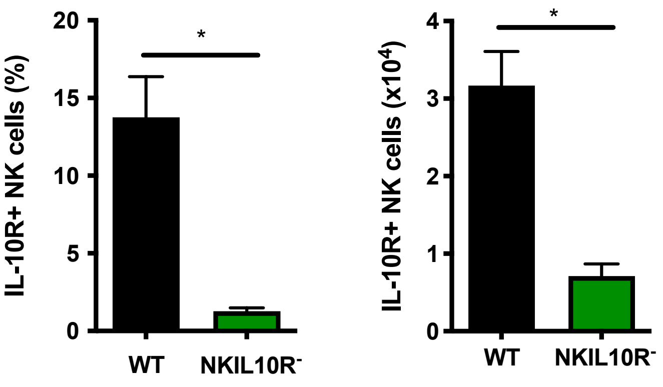

Supplemental Figure Legends:

Supplemental Figure 1.

(A) Expression of p-STAT5 measured by flow cytometry in NK cells (NK1.1+NKp46+TCRβ-) from B6.*il10*$^{-/-}$ naïve mice compared to mice treated with IL-2C or IL-15C i.v. on days 0 and 2, with splenocytes harvested one hour following d2 treatment. Representative histogram and compiled mean fluorescent intensity (MFI) are shown. MFI values normalized relative to naive mice. (B) Percentage and number of NK cells (NK1.1+NKp46+TCRβ-) from IL-10-GFP reporter mice treated with IL-15C i.v. on days 0 and 2 and either STAT3 inhibitor (NSC 74859) or DMSO i.p. on days 0, 2 and 3. Splenocytes were harvested on day 5 for analysis of IL-10-GFP expression. (C) Frequency of NK cells (NK1.1+NKp46+TCRβ-) in CD11b and CD27 maturation groups from B6.*Il10*-GFP reporter (Vert-X) mice treated with IL-15C and either STAT3 inhibitor (NSC 74859) or DMSO as in (B). (D) Percentage and number of NK cells (NK1.1+CD3-) and NKp46+ NK cells from total splenocytes isolated from WT, NKSTAT3^{-}, or B6.*il10*$^{-/-}$ naïve mice. (E) Percentage of NK cells (NK1.1+CD3-) in CD11b and CD27 maturation groups from WT or NKSTAT3^{-}naïve mice. (F) Percentage and number of NK cells (NK1.1+CD3-) from total splenocytes isolated from WT untreated mice compared to WT or NKSTAT3^{-} mice treated with IL-15C i.v. at days -5 and -3. (G) Percentage and number of NK (NK1.1+CD3-) cells and NKp46+ NK cells from total splenocytes isolated from WT, NKSTAT3^{-}, or B6.*il10*$^{-/-}$ mice at 72 hpi with 10^4 Lm i.v. (H) Percentage of GFP+ (IL-10+) cells that are CD49b+/- in the spleen or liver of B6.*Il10*-GFP reporter (Tiger) mice 72 hpi with 10^4 Lm i.v.. GFP+ (IL-10+) cells were gated from total NK1.1+CD3- population. (I) Percentage of CD49b+ or CD49b- cells that are GFP+ (IL-10+) in the spleen or liver from mice as in (H). CD49b+/- cells were gated from total NK1.1+CD3- population.

Supplemental Figure 2.

(A) Immunoblot detection of p-STAT3 (Y705) and total STAT3 from lysates of purified NK cells isolated from the spleens of WT naïve mice following stimulation with rIL-15 (100 pg/mL) for 15 min. Densitometry shown as Arbitrary Units (AU) normalized to β-actin loading controls. (B) Percentage and number of NK cells (NK1.1+CD3-) and NKp46+ NK cells from total splenocytes isolated from naïve WT or NKIL15Ra^{-} mice. (C) Percentage of NK cells in CD11b and CD27 maturation groups in WT or NKIL15Ra^{-} naïve mice. (D) Percentage and number of NK cells and NKp46+ NK cells from total splenocytes isolated from WT or NKIL15Ra^{-} mice at either 24 hpi (D) or 72 hpi with 10^4 Lm i.v. (E). (F) Immunoblot detection of p-STAT3 (Y705) and total STAT3 from lysates of purified NK cells isolated from WT or NKIL15Ra^{-} mice isolated at 72 hpi with 10^4 Lm i.v.. Densitometry shown as Arbitrary Units (AU) normalized to β-actin loading controls.

Supplemental Figure 3.

(A) Percentage and number of NK cells and NKp46+ NK cells from total splenocytes isolated from WT or NKIL10R^{-} naïve mice. (B) Percentage of NK cells (NK1.1+CD3-) in CD11b and CD27 maturation groups in WT or NKIL10R^{-}naïve mice. (C) Percentage and number of NK cells and NKp46+ NK cells from total

splenocytes isolated from WT or NKIL10R$^-$ naïve mice 72 hpi with 10^4 Lm i.v. (D) Percentage and number of IL-10Rα+ NK cells from WT or NKIL10R$^-$ mice at 72 hpi. with 10^4 Lm i.v.
